# Analgesic, Anti-Inflammatory, and GC-MS Studies on *Castanospermum australe* A. Cunn. & C. Fraser *ex* Hook.

**DOI:** 10.1155/2014/587807

**Published:** 2014-02-02

**Authors:** Thankarajan Sajeesh, Thangaraj Parimelazhagan

**Affiliations:** Bioprospecting Laboratory, Department of Botany, Bharathiar University, Coimbatore,Tamil Nadu 641 046, India

## Abstract

The present study was aimed to evaluate the analgesic and anti-inflammatory properties of *Castanospermum australe* and to profile phytochemicals by GC-MS. The ethanolic extracts were prepared by successive solvent extraction using Soxhlet apparatus. The analgesic activity was analyzed by hot plate method and acetic acid-induced writhing test whereas anti-inflammatory study was done by carrageenan induced paw oedema model. The acute toxicity study revealed that ethanol extracts of leaf and bark of *C. australe* were safe even at a higher dose of 2000 mg/kg whereas ethanol extract of seed was toxic at the same dose. In both hot plate method (5.85 s) and acetic acid-induced writhing test (57%), the leaf ethanol extract exhibited significant analgesic activity (*P* < 0.001) at a dose of 400 mg/kg. The anti-inflammatory activity of leaf extract was exhibited by the reduction in paw linear diameter by 64.76% at 400 mg/kg in carrageenan induced paw oedema. The GC-MS analysis of the ethanol extract of leaf revealed sixteen major compounds of which 1,7-dimethyl-4,10-dioxa-1,7-diazacyclododecane, (+)-N-methylephedrine, and permethylspermine were found to be pharmaceutically and the most important. These findings justify that *C. australe* can be a valuable natural analgesic and anti-inflammatory source which seemed to provide potential phytotherapeutics against various ailments.

## 1. Introduction

Medicinal plants are the greatest asset to human health and represent a viable treasure for discovering new potential compounds with various therapeutic effects [1]. In addition, factors such as the availability, affordability, and accessibility of medicinal plants have led to their high demand and usage [2]. Secondary metabolites such as alkaloids, iridoids, and phenolics generally produced by plants, especially for their defense mechanisms, have been implicated in the therapeutic properties of most medicinal plants [3].

Nonsteroidal anti-inflammatory drugs (NSAIDs) are useful for the treatment of mild to moderate acute pain and they also exhibit anti-inflammatory effects by inhibiting the cyclooxygenase enzyme [4]. Most clinically important medicines are steroidal or nonsteroidal anti-inflammatory chemical therapeutics, for treatment of inflammation related diseases including arthritis, asthma, and cardiovascular diseases. Though they possess potent activity, long-term administration is required for the treatment of chronic disease. Besides, these drugs carry different side-effect risks, including gastrointestinal (GI), cardiovascular, and renal complications. Therefore, naturally occurring agents with reduced side effects are required to substitute the chemical therapeutics [5]. However, there is an urgent need to rationalize the system by actually isolating the active constituents and in turn their phytochemical and pharmacological assessment for a better health care system. Furthermore, exploring new phytochemicals from trees, especially from their leaves, offers lesser opportunity for overexploitation and elimination of important sources of phytotherapeutics which are the major concerns in recent years.

The genus *Castanospermum* belongs to the family Fabaceae and has only one species, *Castanospermum australe *A. Cunn. & C. Fraser *ex* Hook., commonly referred to as the Blackbean or the Moreton Bay Chestnut. It is a medicinal as well as toxic plant which is indigenous to Australia and can also be seen in various parts of India, Pakistan, Sri Lanka, and so forth. The seeds of *C. australe *contain an alkaloid castanospermine and are known to be toxic to animals, especially horses, and have on occasion been responsible for human poisonings, although it becomes edible when carefully prepared by pounding into flour, leaching with water, and roasting [6, 7]. As an inhibitor of acid *α*-glucosidase, it has potential use for the development of an animal model of Pompe's disease, a genetic disorder in humans, classified as a lysosomal storage disease, characterized by accumulation of glycogen, especially in muscle tissue [8]. However, castanospermine possesses *α* and *β*-glucosidase inhibitory activities [9] and is showing promise in combating cancer and acquired immunodeficiency syndrome (AIDS) [10–12]. Rhinehart et al. reported that castanospermine (CS) is a potent but nonselective inhibitor of many glycohydrolases including the intestinal disaccharidases [13]. The antiparasitic activity of castanospermine was demonstrated by Wright et al., preventing adhesion of *Plasmodium falciparum* to infected erythrocytes [14]. Although antiviral activity appears to offer the most general therapeutic potential, castanospermine has also been shown to be immunosuppressive, promoting heart and renal allograft survival in rats [15]. Moreover, castanospermine reduced the blood glucose level after injection at 150 *μ*M/kg i.p. [16]. More recently, castanospermine has been shown to be effective *in vitro* against all four serotypes of dengue fever virus and to prevent mortality of mice infected with the virus at doses as low as 10 mg/kg bodyweight/day [17, 18].

As an attempt to evaluate the pharmacological activities, Ahmed isolated saponins from leaf of *C. australe* and reported their analgesic activity [19]. In search for new bioactive carbohydrate-like compounds, Jones et al. reported the isolation of the novel alkaloid, 1-epialexine, from the leaves and stems of *C. australe* [20]. It is evident from the fact that the various constituents of *C. australe*, for example, alkaloid, saponin, and flavonoid, could exhibit biological activity to the various extents and these could be considered as promising compounds for clinical utilization. In the present study, attempt has been made to scientifically validate the analgesic and anti-inflammatory properties of the ethanol extract of *C. australe* and to explore the phytoconstituents holding the biological properties.

## 2. Materials and Methods

### 2.1. Plant Materials

The fresh leaves and bark were collected during the month of January 2011 and seeds during the month of June 2011 from the Botanical Garden of Mar Ivanios College, Thiruvananthapuram, Kerala, India. The taxonomic authentication of the plant was done from the Botanical survey of India, Southern Regional Centre, Coimbatore, Tamil Nadu (number 791). The surface pollutants were removed by thorough washing of fresh materials using distilled water and were shade-dried at room temperature. The dried leaf, bark, and seed were powdered using mixer and were used for solvent extraction.

### 2.2. Extract Preparation

The powdered plant materials such as leaf, bark, and seed were extracted separately with petroleum ether, benzene, and ethanol successively in the increasing order of their polarity using Soxhlet apparatus. The petroleum ether and benzene help to remove lipid constituents, chlorophylls, and other nonpolar substances which will reduce the biological activities. Each time before extracting with the next solvent, the thimble was dried in a hot air oven at 40°C. The ethanol extracts were then concentrated using rotary vacuum evaporator and were used for *in vivo* studies.

### 2.3. Chemicals and Standard Drugs

The standard drugs such as aspirin, pentazocine, and indomethacin were purchased from Sigma-Aldrich (Bengaluru branch, Karnataka, India). All other chemicals like carrageenan, carboxymethyl cellulose, and so forth were obtained from Himedia (Mumbai, Maharashtra, India) and of the highest purity and analytical grade.

### 2.4. Animals and Ethics

Swiss albino mice (20–30 g) and Wistar albino rats (200–250 g) were used for the animal studies and were carried out at Nandha College of Pharmacy, Erode, Tamil Nadu. The animals were maintained under standard conditions of temperature (24 ± 1°C), relative humidity (55 ± 1%), and light/dark cycles (12/12 h) and were fed with standard diet and water *ad libitum*. All animals were acclimatized for at least 1 week and were fasted for 12 h (free access to water but not to food) before the beginning of experiments. All the animal experimental protocols were subjected to Institutional Animal Ethical Committee for getting permission and clearance (688/2/C-CPCSEA/2011/12) to conduct the same.

### 2.5. Acute Oral Toxicity

The acute oral toxicity studies were carried out by fixed dose procedure according to the Organization for Economic Co-operation and Development (OECD) guideline 420 with slight modifications [21]. For both sighting study and main study, healthy female Swiss albino mice weighing between 20 and 30 g were selected by random sampling technique, since female mice exhibit much behavioral changes due to hormonal action rather than male mice. They were housed in different plastic cages and maintained at room temperature, photoperiod of 12 h, and frequent air changes. They had free access to only water for a short fasting period before the treatment with the plant extract. The sighting study provides the data for the selection of the appropriate starting dose for the main study. The test substance was administered to single animal in a sequential manner and was observed thoroughly for at least 24 hours. The starting dose for the sighting study was selected from the fixed dose levels of 5, 50, 300, and 2000 mg/kg as a dose expected to produce evident toxicity based, when possible, on evidence from *in vitro* and *in vivo* data from the same extract and from related phytochemicals. In the absence of such information, the starting dose will be 300 mg/kg. Based on the phytochemical evaluation, the *C. australe* extracts were administered at a dose of 2000 mg/kg body weight by oral gavage in the sighting study.

However, for main study, the mice were divided into three groups of 5 animals each in which two groups received leaf and bark extracts at dose of 2000 mg/kg whereas the third group received seed extract at 1000 mg/kg. The animals were observed for general behavioral changes and signs of toxicity and mortality such as alertness, grooming, touch response, pain response, tremors, convulsions, righting reflex, gripping strength, pinna reflex, corneal reflex, pupils, urination, salivation, skin colour, lacrimation, and hyperactivity continuously for 1 h after treatment, then intermittently for 4 h, and thereafter over a period of 24 h. This method requires short period and thus is used to fix the doses for simpler animal studies rather than finding lethal dose (LD_50_).

### 2.6. Analgesic Activity

#### 2.6.1. Hot Plate Method/Centrally Mediated Analgesic Activity

The hot plate test which postulates the assessment of centrally mediated analgesic effect was performed by the method described by Lanhers et al. with slight modifications [22]. The male mice were divided into eight groups of six animals each. The temperature of the hot plate in analgesiometer was regulated to 55 ± 1°C. Each mouse was placed on the hot plate in order to obtain the animal's responses to electrical heat induced nociceptive pain stimulus. The time taken for each mouse to jump out of the chamber (i.e., reaction time) was noted and recorded in seconds. Each mouse served as its own control and the “initial reaction time” was determined before treatment. The group 1 (control group) animals received only the vehicle (carboxymethyl cellulose, 10 mL/kg, p.o.) whereas group 2 (reference group) animals were administered with pentazocine at a dose of 30 mg/kg, i.p. The remaining six groups accordingly received with leaves (groups 3 and 4) and bark (groups 5 and 6) ethanol extracts at doses of 200 and 400 mg/kg, p.o., and seed (groups 7 and 8) ethanol extract at doses of 100 and 200 mg/kg, p.o. Latency to exhibit antinociceptive responses such as licking of the forepaws and eventually jumping out of the chamber was determined at 30, 60, 120, and 240 minutes after administration of the plant extracts and reference drug.

#### 2.6.2. Acetic Acid-Induced Writhing Response/Peripherally Mediated Analgesic Activity

The study was carried out using the method described by Collier et al. that will support the preferential evaluation of possible peripheral analgesic effects of ethanolic extracts of *C. australe* [23]. Initially, Swiss albino male mice weighing 20–30 g were divided manually into eight groups of six animals each and were fasted for 12 h prior to the experiment. The writhing response was elicited by an intraperitoneal injection of 0.75% acetic acid at the dose of 0.1 mL/10 g body weight to all the groups of mice. The first group was considered to be control group which received only the vehicle, carboxymethyl cellulose (10 mL/kg, p.o.). The second group was administered with the standard analgesic drug aspirin at a dose of 100 mg/kg. The ethanolic extracts of leaves (groups 3 and 4), bark (groups 5 and 6), and seed (groups 7 and 8) were administered orally along with the vehicle, carboxy methyl cellulose, 1 h before the acetic acid injection to the remaining six groups at two doses each to make it available to the tissues of the body. Based on the acute toxicity studies, the doses for leaves and bark extracts were decided to be 200 and 400 mg/kg whereas those of seed was 100 and 200 mg/kg. The number of writhes was counted for 15 min beginning from 5 min after the acetic acid injection. Percentage of inhibition of writhing response was also calculated and compared with that of animals in the control as well as reference group.

### 2.7. Acute Anti-Inflammatory Activity

#### 2.7.1. Carrageenan Induced Paw Oedema in Rats

The Wistar albino male rats weighing 200–250 g were divided into eight groups, each consisting of six animals and were fasted overnight. Paw oedema was induced in all the groups of animals by subplantar injection of 0.1 mL of freshly prepared 1% carrageenan suspension (in 1% carboxymethyl cellulose) into the right hind paw of each rat [24]. The test drug and ethanolic extracts of leaf, bark, and seed (suspended in 1% CMC) were administered orally 1 h before the injection of carrageenan in order to increase their availability to the acute inflammation site. The first group was assigned to be the control group which received only the vehicle, carboxymethyl cellulose (10 mL/kg, p.o.), whereas the second group was administered with the anti-inflammatory drug, indomethacin, at a dose of 10 mg/kg, i.p. for comparative pharmacological assessment. The remaining six groups were administered with leaves (groups 3 and 4) and bark (groups 5 and 6) ethanol extracts at doses of 200 and 400 mg/kg, p.o., and seed (groups 7 and 8) ethanol extract at doses of 100 and 200 mg/kg, p.o. The linear diameter of the injected paw was measured with the digital vernier caliper (Model number CD-6′′CSX, Mitutoyo Digimatic Caliper, Japan) before and at 60, 120, 180, and 240 min intervals after the injection of carrageenan [25]. The relative potency of the drugs/plant extracts under investigations was compared with the activities at different intervals.

### 2.8. Gas Chromatography-Mass Spectrometry Analysis

GC-MS analysis was done using Clarus 600 gas chromatograph system equipped with Clarus 600 C mass spectrometer (PerkinElmer precisely, USA). An Elite-5MS fused silica capillary column coated with a 5% diphenyl/95% dimethylpolysiloxane stationary phase (60 m × 0.25 mm, film thickness 0.10 *μ*m; PerkinElmer precisely, USA) was used for gas chromatography. The injector temperature was programmed to 200°C whereas the oven temperature was increased from 70°C to 300°C for the total run time of 35 min. The inert gas helium was used as carrier gas at a flow rate of 1.0 mL/min. Initially, ethanol (blank) only was run from which the solvent delay was fixed to 4 min. The electron ionization mode with ionization energy of 70 eV; ion source temperature of 200°C; GC interface temperature of 240°C; scan interval of 0.2 second, and fragments range from 50 to 600 *m*/*z* were set for the mass spectrometry analysis. Manual injection of 1 *μ*L of the ethanol extract of *C. australe* leaf in a splitless mode was followed. The identification of compounds was done by comparing the mass spectra of the respective peaks obtained in the GC-MS with the mass fragmentation patterns of standards in the National Institute of Standard Technology (NIST) library using the Turbomass software (ver 5.4.0.).

### 2.9. Statistical Analysis

All the experiments were done with groups of six animals each and the results were expressed as mean ± standard error of mean (SEM). The data were statistically analyzed using SPSS version 17.0 by means of one-way ANOVA followed by Dunnett test for analgesic and anti-inflammatory studies.

## 3. Results and Discussion

### 3.1. Extract Yield and Acute Oral Toxicity

Among the different solvents used, extract recovery percent was found to be higher in ethanol and was 22.5, 15.3, and 13.7 g/100 g of dried powder for bark, leaf, and seed, respectively. Then, the ethanol extracts of leaf, bark, and seed of *C. australe* were evaluated for their acute toxicity in mice and the different parameters were observed (Table 1). The leaf and bark extracts did not show any signs of mortality or toxicity in terms of changes in general behaviors even at the highest dose of 2000 mg/kg both in sighting and main studies. On the other hand, the seed extract was found to be toxic at 2000 mg/kg in the sighting study whereas safe at 1000 mg/kg in the main study. Thus, acute toxicity study propose that leaf and bark extracts can be considered as a broad nontoxic one while seed extract cannot. On the basis of acute toxicity study, the doses for further *in vivo* studies were designed to be 200 and 400 mg/kg for leaf and bark extracts whereas 100 and 200 mg/kg for seed extract.

### 3.2. Analgesic Activity

#### 3.2.1. Hot Plate Method/Centrally Mediated Analgesic Activity

Table 2 represents the effects of ethanol extracts of *C. australe* against heat induced pain stimulus in Swiss albino mice. After 120 min, the standard and plant extracts were able to significantly (*P* < 0.001) increase in the reaction time compared to other intervals. The standard pentazocine showed the highest reaction time of 8.64 s after 120 min even at a dose of 30 mg/kg which confirms its effect as centrally mediated analgesic activity. Among the different parts of *C. australe*, the ethanol extract of leaf significantly prolonged the reaction time by withstanding the heat stimulation to the central nervous system at 200 and 400 mg/kg. Moreover, all the extracts of the plant exhibited dose- and time-dependent analgesic activity compared to the control.

Nonsteroidal anti-inflammatory drugs (NSAIDs) are useful for the treatment of mild to moderate acute pain, including musculoskeletal injuries, and can be useful in patients with chronic inflammatory conditions [26]. The evaluation of central analgesic activity through hot plate test selected possesses several advantages such as particularly the sensitivity to strong antinociceptive and limited tissue damage [27]. The mediators—prostaglandins and bradykinin—were suggested to play an important role in analgesia [28]. Moreover, a number of flavonoids and tannins have been reported to produce analgesic activity mediated by central system [29]. Thus, the strong analgesic activity produced by the ethanolic extracts of leaf and seed of *C. australe* may be due to the inhibition of effects or production of prostaglandins and bradykinin. However, the presence of phenolics, flavonoids, or alkaloids in the extracts can enhance the centrally mediated response in a dose-dependent manner. The strong activity of the hygienic ethanol extracts of *C. australe* against the nociceptor response to pain will provide a cost effective and reliable source of analgesic drug.

#### 3.2.2. Acetic Acid-Induced Writhing Response/Peripherally Mediated Analgesic Activity

The peripherally mediated analgesic activities of *C. australe* analyzed by acetic acid-induced writhing response in mice are shown in Table 3. The control mice were found to produce 78.0 writhes after the injection of acetic acid. Among the different parts studied, the ethanol leaf extract showed better analgesic activity where the writhes were reduced to 43.0 and 33.25 at doses of 200 and 400 mg/kg, respectively. The percentage of inhibition for those was comparable with that of standard drug aspirin which showed inhibition of 53% at a dose of 100 mg/kg. The ethanol extract of bark also reduced the number of writhes to 43.75 (44% inhibition) at 400 mg/kg. On the other hand, the extract of seed showed better reduction in the number of writhes compared to the control and were 24% and 41% for 100 and 200 mg/kg, respectively.

The writhing response of the mouse to an intraperitoneal injection of noxious chemical is used to screen both peripherally and centrally acting analgesic activity. Acetic acid causes analgesia by liberating endogenous substances and many others that excite pain nerve endings [30]. However, the writhing induced by chemical substances is due to sensitization of nociceptors by prostaglandins [25]. Moreover, the peripherally acting antinociceptive can be monitored by the abdominal constriction response in mice induced by acetic acid. Aspirin and other NSAIDs can inhibit cyclooxygenase in peripheral tissues, thus, interfering with the mechanism of transduction in primary afferent nociceptors [31]. The significant effect (*P* < 0.001) of the extracts of *C. australe* against the noxious stimulus may be an indication that it depressed the production of irritants and thereby bringing a reduction in the number of writhes in animals. The mechanism of analgesic action of the ethanol extract of leaf and seed could probably be due to the blockade of the effect or the release of endogenous substances that excite pain nerve endings similar to aspirin and NSAIDs. It is also noted that the peripheral effects mediated by the ethanol extracts of *C. australe* in a dose-dependent manner may involve the inhibition of synthesis or action of prostaglandins. Moreover, this study also reveals that the active principles in *C. australe* responsible for such analgesic effect can be phenolics, flavonoids, and so forth rather than saponins since they would have been removed by the low polar solvents during extraction.

### 3.3. Acute Anti-Inflammatory Activity

#### 3.3.1. Carrageenan Induced Paw Oedema in Rats

The anti-inflammatory activities of ethanol extracts of leaves, bark, and seed of *C. australe* against carrageenan induced paw oedema were analyzed and the results are expressed in Table 4. Oral pretreatment of animals with ethanol extracts of leaf and seed resulted in a significant inhibition (*P* < 0.01 and *P* < 0.001) of carrageenan-evoked hind paw edema in a dose- as well as time-dependent manner. The paw linear diameter of control mice (increased by 2.1 mm) suggests that paw oedema persisted even after 4 hours of carrageenan injection. A remarkable reduction in inflammation (1.21 mm) was shown by the ethanol extract of seed at a dose of 200 mg/kg. However, at the end of the forth hour, the inflammation was reduced by 1.36 (64.76%) and 1.1 mm (52.38%) for leaf and bark extracts, respectively, at 400 mg/kg. In case of standard-indomethacin, significant lowering of the paw linear diameter was observed, as the normal paw diameter was reduced to 5.28 mm (reduction by 1.9 mm) even at a lower dose of 10 mg/kg.

Apart from the analgesic effects, NSAIDs can also exhibit anti-inflammatory effects by inhibiting the cyclooxygenase enzyme (COX) [26]. Carrageenan induced hind paw edema is the standard experimental model of acute inflammation in which the paw oedema induced by the subplantar injection of carrageenan in rats is biphasic; the early phase involves the release of serotonin, histamine, and kinins while the late phase is mediated by prostaglandins [28]. In the present study, the ethanol extracts of leaf and seed of *C. australe* exhibited a weak inhibitory effect at early phase but were able to effectively inhibit the increase of paw volume during the late phase (4 hrs after carrageenan injection) of inflammation. Based on this observation and the biphasic nature of carrageenan induced paw edema, it is possible to propose that the significant activity of the *C. australe* extract observed in the last phase of inflammation may be due to the ability of the extract to inhibit the release and/or activity of the late mediators involved in carrageenan induced paw oedema. Moreover, it is also reported that the analgesic and anti-inflammatory activities were exerted by many flavonoids, terpenoids, sterols, saponins, and so forth [32, 33]. Therefore, the analgesic and anti-inflammatory properties exhibited by the ethanol extracts in the present study may be due to the individual or synergistic action of various phytochemical compounds in *C. australe*.

### 3.4. Gas Chromatography-Mass Spectrometry Analysis

**Figure 1:**
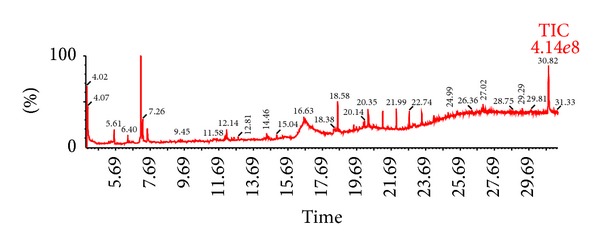
GC-MS chromatogram of ethanol extract of *C. australe* leaf.

The ethanol extract of leaf which showed remarkable analgesic and anti-inflammatory effects was analyzed by GC-MS and the chromatogram is shown in Figure 1. The relative retention times (Rt) and mass spectra of the extract components were compared with those of authentic samples and with mass spectra from the NIST library. As shown in Table 5, GC-MS analysis of all parts resulted in the identification of sixteen major compounds, with more than 90% similarity with the standard mass spectra in the library. Among them, compounds present in higher percentages were allyl n-octyl ether, 2-methyl-7-azabicyclo[4.1.0]heptanes, 1-(4-bromo-phenyl)-3-(4-hydroxy-butylamino)-pyrrolidine-2,5-dione, methyl-2-O-methyl-*α*-D-glucopyranosiduronic acid methyl ester, 1,7-dimethyl-4,10-dioxa-1,7-diazacyclododecane, 1-[[3-[dimethylamino]propyl]imino]-1,3,4,10-tetrahydro-7-(trifluoromethyl)-9(2H)-acridinone, N′-[3-(dimethylamino)propyl]-N,N-dimethyl-1,3-propanediamine, 2-acetyl-2,3,5,6-tetrahydro-1,4-thiazine, (+)-N-methylephedrine, and so forth. However, the peaks with retention times such as 6.40 (0.38%), 7.26 (0.98%), 12.81 (0.13%), 15.04 (0.25%), 24.17 (0.48%), 27.02 (0.67%), and 30.82 (5.62%) did not provide any hits in the NIST library search.

Even though the separation and identification of phytochemicals by GC-MS has mostly been attempted to compounds in the essential oils of herbs [34], the chemical profile having pharmacological activities can be detected. In addition to that, GC-MS analysis revealed many pharmacologically important phytochemicals in the ethanol extract of *C. australe *leaf. Oxazine derivatives have been used in the treatment of neurological disorders and they also possess *in vitro* antineoplastic potential [35]. The 4-thiazine derivatives have been reported to have antitubercular activity and an efficient inhibitor of IL-8 production by Human gingival fibroblast (HGF) and nitric acid production by activated RAW 2647 cells [36, 37]. Allyl n-octyl ether has antibacterial activity and is used as pharmaceutical intermediates [38]. N-Methylephedrine is a direct acting sympathomimetic agent with pronounced effect on *α* and *β*-adrenergic receptors and has stimulating effects on the central nervous system. It has more prolonged though less potent action than adrenaline. It is used to relieve asthma and bronchitis and as resolving agent [39]. Chloroacetic acid is used as an indirect additive in food contact substances, herbicide, preservative, and bacteriostat. Uronic acids are reducing sugars of biological relevance. Their derivatives are poor donors, due to the deactivating effect of the electron-withdrawing carboxylate group, and are involved in the metabolism of many drugs and endogenous compounds [40].

Furthermore, spiroheterocyclic pyrrolidine dione derivatives are widely used as pesticides. Pyrrolidine also known as tetrahydropyrrole—an organic compound found naturally in the plants—is used as pharmaceutical drugs such as procyclidine and bepridil (anticholinergic) for the treatment of Parkinson's disease, acute dystonia, and akathisia syndrome [41]. N,N-Dimethyl-1,3-propanediamine has been applied in hair care products, betaine, antistatic agents intermediates, and so forth. Moreover, patent has been filed for substituted azabicyclo[4.1.0.]heptane compounds for use as monoamine reuptake inhibitors and pharmaceutically acceptable salts. In addition to that, 1,7-dioxa-4,10-diazacyclododecane exhibits DNA binding and cytotoxic activity which in turn lights up its broad biological activities [42]. 2-Amino-2-methyl-1-propanol can be used to study biological buffers and to make surface active agents, pharmaceuticals, and so forth. Moreover, it is used as an emulsifying agent for cosmetic creams and is used in hair sprays, hair dyes, Pamabrom (drug), and absorbents for acidic gases. Gao et al. synthesized a novel series of 10-benzyl-9(10H)-acridinones and tested for their *in vitro* antitumor activities against CCRF-CEM cells [43]. Moreover, there has been renewed interest in the antimalarial activity of acridines and their congeners, the acridinones [44]. Spermine is found in a wide variety of organisms and tissues and it possesses antibiotic, antioxidant, and anti-inflammatory properties [45, 46]. Moreover, it is also reported that spermine analogues were recognized to produce high inhibitory effect on cell growth [47, 48]. In addition to that, the unidentified compounds with higher area percentages may also contribute to the biological properties of the plant to some extent. These peaks can be new compounds with potential activities which have to be screened further. Thus, from these findings, it can be suggested that apart from saponins in the nonpolar extracts [19], compounds like 1,7-dimethyl-4,10-dioxa-1,7-diazacyclododecane, (+)-N-methylephedrine, and permethylspermine in the ethanol extract of* C. australe* leaf can also be responsible for the analgesic and anti-inflammatory effects which may be mediated by their synergistic action.

## 4. Conclusions

The growing demand to replace synthetic drugs or additives by natural ones has increased enormous interest on the exploration of analgesic and anti-inflammatory properties of plants in both academia and industry. The present findings justify that *Castanospermum australe* can be used as a natural source for alternative or supplementary therapeutic drug for the treatment of analgesic and inflammatory diseases. Further, investigation dealing with isolation and mechanism of action of active components in ethanol extract is warranted for the possible development of new class of cost effective drug.

## Figures and Tables

**Table 1:** Behavioral changes during the main study for acute oral toxicity analyses of *C*.  *australe* ethanol extracts.

Parameter	Leaf (2000 mg/kg)		Bark (2000 mg/kg)		Seed (1000 mg/kg)	
	Before	After 24 h	Before	After 24 h	Before	After 24 h
Alertness	N	N	N	N	N	N
Grooming	N	N	N	N	N	N
Touch response	P	P	P	P	P	P
Pain response	P	P	P	P	P	P
Tremors	N	N	N	N	N	N
Convulsions	A	A	A	A	A	N
Righting reflex	N	N	N	N	N	N
Gripping strength	N	N	N	N	N	N
Pinna reflex	N	N	N	N	N	N
Corneal reflex	N	N	N	N	N	N
Pupils	N	N	N	N	N	N
Urination	N	N	N	N	N	N
Salivation	A	A	A	A	A	A
Skin colour	N	N	N	N	N	N
Lacrimation	A	A	A	A	A	A
Hyper activity	A	A	A	A	A	A
Mortality	A	A	A	A	A	A

N: normal, P: present, and A: absent.

Each group contains five animals and the parameters were observed in ≥4 animals.

**Table 2:** Analgesic activities of ethanol extracts of *C*.  *australe* using hot plate method.

Groups	Dose (mg/kg)	Reaction time (seconds)				
		Initial	Time after drug administration (minutes)			
			30	60	120	240
Control	—	1.94 ± 0.20	2.13 ± 0.16	2.02 ± 0.21	1.92 ± 0.17	2.26 ± 0.31
Pentazocine	30	2.36 ± 0.18	5.18 ± 0.13*	6.25 ± 0.15*	8.64 ± 0.12*	7.98 ± 0.09*
Leaf	200	1.75 ± 0.25	3.25 ± 0.25*	4.52 ± 0.23*	5.75 ± 0.25*	5.50 ± 0.16*
	400	2.25 ± 0.25	3.95 ± 0.17*	5.75 ± 0.16*	6.59 ± 0.21*	5.85 ± 0.20*
Bark	200	2.13 ± 0.21	2.97 ± 0.15	3.68 ± 0.19*	4.97 ± 0.18*	4.12 ± 0.21*
	400	1.93 ± 0.18	3.12 ± 0.20*	4.38 ± 0.12*	5.54 ± 0.14*	4.89 ± 0.19*
Seed	100	2.10 ± 0.19	3.15 ± 0.16*	4.11 ± 0.20*	5.06 ± 0.21*	4.63 ± 0.13*
	200	2.24 ± 0.13	3.86 ± 0.21*	4.79 ± 0.22*	5.58 ± 0.18*	4.51 ± 0.14*

Values are mean of observations from six mice (*n* = 6) ± SEM.

Significantly different from control at *P < 0.001.

**Table 3:** Acetic acid-induced writhing response of ethanol extracts of *C*.  *australe* in mice.

Group	Dose (mg/kg)	Number of writhes	Inhibition (%)
Control	—	78.0 ± 1.08	—
Aspirin	100	36.25 ± 2.06*	53
Leaf	200	43.0 ± 1.83*	45
	400	33.25 ± 1.38*	57
Bark	200	62.0 ± 1.29*	21
	400	43.75 ± 1.38*	44
Seed	100	59.50 ± 1.71*	24
	200	45.75 ± 1.89*	41

Values are mean of observations from six mice (*n* = 6) ± SEM.

Significantly different from control at *P < 0.001.

**Table 4:** Anti-inflammatory effect of ethanol extracts of *C*.  *australe *on carrageenan induced paw oedema in rats.

Groups	Dose (mg/kg)	Oedema induced by carrageenan (mm)				
		0 hr	1 hr	2 hrs	3 hrs	4 hrs
Control	—	4.86 ± 0.32	5.49 ± 0.11	5.92 ± 0.32	6.53 ± 0.18	6.96 ± 0.21
Indomethacin	10	5.08 ± 0.17	5.21 ± 0.15	5.26 ± 0.12	5.44 ± 0.10**	5.28 ± 0.19**
Leaf	200	4.92 ± 0.06	5.47 ± 0.36	5.32 ± 0.38	5.85 ± 0.15*	6.30 ± 0.16*
	400	5.11 ± 0.22	5.45 ± 0.20	5.25 ± 0.29	5.57 ± 0.21**	5.85 ± 0.12**
Bark	200	4.98 ± 0.10	5.53 ± 0.16	5.48 ± 0.19	5.92 ± 0.19*	6.25 ± 0.11*
	400	4.96 ± 0.14	5.34 ± 0.20	5.36 ± 0.12	5.67 ± 0.13**	5.96 ± 0.18**
Seed	100	4.93 ± 0.25	5.73 ± 0.23	5.30 ± 0.23	6.01 ± 0.13	6.13 ± 0.16**
	200	5.04 ± 0.51	5.48 ± 0.12	5.35 ± 0.18	5.87 ± 0.17*	5.93 ± 0.15**

Values are mean of observations from six rats (*n* = 6) ± SEM.

Significantly different from control at *P < 0.01 and **P < 0.001.

**Table 5:** Chemical profile identified by GC-MS analysis of ethanol extract of *C*. * australe* leaf.

S. number	RT	Compound	Area percentage
1	4.07	6-(4-Chlorophenyl)tetrahydro-2-methyl-2H-1,2-oxazine	0.39
2	5.61	2-Acetyl-2,3,5,6-tetrahydro-1,4-thiazine	0.81
3	7.15	Allyl n-octyl ether	4.24
4	7.53	(+)-N-Methylephedrine	0.78
5	9.45	N′-[3-(Dimethylamino)propyl]-N,N-dimethyl-1,3-propanediamine	0.12
6	12.14	Methyl-4-O-methyl-*β*-D-xylopyranoside	0.70
7	14.46	Chloroacetic acid tridecyl ester	0.76
8	16.63	Methyl-2-O-methyl-*α*-D-glucopyranosiduronic acid methyl ester	1.82
9	18.58	1-(4-Bromo-phenyl)-3-(4-hydroxy-butylamino)-pyrrolidine -2,5-dione	2.09
10	19.52	N′-[3-(dimethylamino)propyl]-N,N-dimethyl-1,3-propanediamine	0.92
11	20.14	2-Methyl-7-azabicyclo[4.1.0]heptane	2.21
12	20.35	1,7-Dimethyl-4,10-dioxa-1,7-diazacyclododecane	1.79
13	21.20	1-[[3-[Dimethylamino]propyl]imino]-1,3,4,10-tetrahydro-7-(trifluoromethyl)-9(2H)-acridinone	0.97
14	21.99	Permethylspermine	0.69
15	22.74	S-[2-[N,N-Dimethylamino]ethylpyrollidine-N-carbonylthiocarbohydroximate	0.58
16	23.47	2-Amino-2-methyl-1-propanol	0.40

RT: retention time.
